# Relationship Between Evolutionary Diversity and Aboveground Biomass During 150 Years of Natural Vegetation Regeneration in Temperate China

**DOI:** 10.1002/ece3.70390

**Published:** 2024-10-08

**Authors:** Qilong Tian, Xiaoping Zhang, Miaoqian Wang, Jie He, Xiaoming Xu, Liang He, Haijie Yi, Haojia Wang

**Affiliations:** ^1^ College of Ecology and Environment, Key Laboratory of Oasis Ecology of Education Ministry Xinjiang University Urumqi China; ^2^ Institute of Soil and Water Conservation, State Key Laboratory of Soil Erosion and Dryland Farming on Loess Plateau Chinese Academy of Sciences and Ministry of Water Resources Yangling China; ^3^ University of Chinese Academy of Sciences Beijing China; ^4^ Institute of Soil and Water Conservation Northwest A&F University Yangling China; ^5^ College of Urban, Rural Planning and Architectural Engineering Shangluo University Shangluo China

**Keywords:** carbon sink, ecosystem function, phylogenetic diversity, phylogenetic structure, vegetation succession

## Abstract

While the link between plant species diversity and biomass has been well‐studied, the impact of evolutionary diversity on community biomass across long timescales or ongoing change remains a subject of debate. We elucidated the association between evolutionary diversity and community aboveground biomass (AGB) using an ideal experimental system with over 150‐year history of natural vegetation regeneration. Higher phylogenetic diversity facilitated the sampling effect under the influence of environmental filtering, and caused an increase in AGB. Phylogenetic structure varied from aggregation to dispersion during the later period of vegetation recovery (70–150 years), which was correlated with increases in niche complementarity and increasing AGB. Woody plant evolutionary diversity was used as a key to predict the relationship between vegetation recovery and AGB, with a total explanatory power of ~84.7%. Mixed forests composed of evergreen conifers and deciduous broadleaf forests had higher carbon sequestration potential than that of pure forests, which is advantageous for increasing top‐stage AGB. This research expands our knowledge of the causes and effects of biodiversity and ecosystem function dynamics over time and space, which is important for accurately predicting future climate change effects.

## Graphical abstract

The research revealed the association between evolutionary diversity and community AGB during long‐term vegetation restoration in the temperate zone using an ideal experimental system with over 150 years of natural vegetation restoration. This is essential for elucidating the link between biodiversity and ecological functions in the context of climate change.

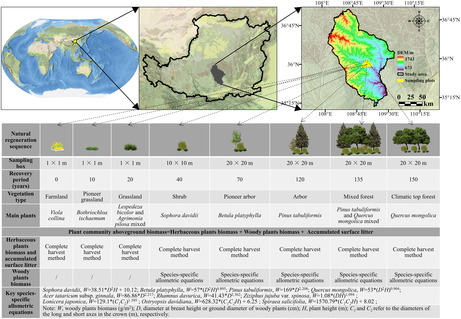

## 1 Introduction

The relationship between biodiversity and ecological function is a pivotal challenge confronting ecosystems (Fraser et al. 2015; Grace et al. 2016; Craven et al. 2018). In forests, ecosystem aboveground components, litter, belowground components, and soil are four major sources that are responsible for nutrient flux (Persch et al. 2015; Awasthi, Bargali, Bargali, Khatri, and Jhariya 2022, Awasthi, Bargali, Bargali, and Khatri 2022). Among them, belowground parts contribute > 30% of net primary productivity of the terrestrial ecosystems (Karki et al. 2017; Mikieleko et al. 2021; Pandey et al. 2024). Aboveground biomass (AGB) represents the energy acquisition capacity of an ecosystem, therefore serving as a crucial indicator of ecosystem structure and function (Yuan et al. 2021; Awasthi, Bargali, Bargali, and Jhariya 2022). AGB plays a vital role in terrestrial ecosystem material cycling and climate change processes, including carbon cycling, soil nutrient allocation, biomass‐driven structural alterations, and habitat structuring (Houghton, Hall, and Goetz 2009; Liu, Xu, et al. 2021; Kröel‐Dulay et al. 2022). Understanding the link between plant diversity and AGB is essential for sustaining long‐term ecosystem function (Mori et al. 2021). For example, the rise of CO_2_‐based greenhouse gas emissions from 280 to 400 ppm since the preindustrial era has raised concerns about vegetative carbon absorption (McDowell et al. 2020). Pan et al. (2011) determined that the global forest biome annually sequesters ~861 ± 66 PgC, with living biomass (including the AGB pool) accounting for ~42%. This underscores the substantial potential of AGBs in absorbing and sequestering atmospheric carbon (Wang and Ali 2021) and emphasizes the importance of addressing AGB and soil–plant feedbacks (Lewis et al. 2019). Recognizing the potential of tree restoration for climate change mitigation (Khanalizadeh et al. 2023) is crucial alongside additional measures to enhance vegetative carbon sink capacity (IPCC reports 2022, 2023; Friedlingstein et al. 2019).

Large carbon sink gains through plant diversity restoration are anticipated to alleviate the climate change crisis (Ramachandra and Bharath 2020). Extensive research has indicated a correlation between carbon stocks and plant diversity recovery (Ferreira et al. 2018; Bongers et al. 2021; Schnabel et al. 2021; Sandoval‐Calderon et al. 2024), primarily influenced by selection impact among species and niche complementarity mechanisms (Loreau and Hector 2001). The niche complementing theory (also known as facilitation) posits that high‐diversity groups use resources more efficiently, exhibiting more functions (e.g., carbon sink) compared with those of low‐diversity groups (Tilman, Lehman, and Thomson 1997; Tilman 1999). The sampling effect or “mass‐ratio” hypothesis states that greater species diversity increases the probability of harboring dominant species and ecosystem functions (e.g., carbon sink) as sampling progresses (Grime 1998). Previous studies have found that increasing biodiversity enhances essential ecological functions (Mazzochini et al. 2019; Jochum et al. 2020; Rahman et al. 2021).

The relationship between biodiversity and ecosystem functions (BEF) has been the subject of several previous studies, with particular emphasis on taxonomic diversity encompassing abundance and richness measures (Liu, Zhang et al. 2021, 2022; Pan et al. 2022). This concept assumes that all species within a community are equal (Li et al. 2018; Zhao et al. 2020), despite the fact that plant communities are the product of a complex interplay between environmental and evolutionary factors (Ricklefs 2006; Yue and Li 2021). Therefore, phylogeny is more commonly used in community ecology (Webb et al. 2002; Molina‐Venegas et al. 2021; Gaüzère et al. 2022). Research on evolutionary variations in lineages within a community using genomic information (Eiserhardt, Couvreur, and Baker 2017; Tucker et al. 2017) defines phylogenetic diversity as the evolutionary history of a studied community (Webb 2000). Therefore, phylogenetic diversity can be used to differentiate between closely related and distantly related species and provide an objective response to patterns of diversity, functional characteristics, and community composition (Srivastava et al. 2012; Mahayani et al. 2020; Staab et al. 2021). In addition, phylogenetic diversity can be used to predict ecosystem functions and has greater explanatory power for AGB than previously speculated (Cadotte, Cardinale, and Oakley 2008; Flynn et al. 2011; Luo et al. 2019). Previous studies have observed that the mechanisms governing BEF can exhibit temporal variations (Mouquet, Moore, and Loreau 2002; Lasky et al. 2014; Huang et al. 2018). Thus, studies on BEF dynamics should incorporate long‐term timescales or observe continuous changes in BEF relationships, particularly in understudied temperate regions.

To further our understanding of BEF dynamics in temperate regions, we conducted a study at a natural test site in the Ziwuling area of the Loess Plateau, China. The area is situated within the temperate zone and has experienced over 150 years of natural vegetation regeneration processes. Due to the rapid shifts in species composition and ecosystem functions across various recovery periods, this site provides an ideal system for observing the dynamics of BEF interactions (Parker 1997; Letcher and Chazdon 2009; Khorchani et al. 2022). Employing the “space‐for‐time” approach (Blois et al. 2013), we assessed the evolutionary diversity and AGB characteristics of different stages of natural vegetation regeneration. Our study aimed to address the following research questions: (1) How does vegetation regeneration affect AGB, phylogenetic diversity, and phylogenetic structure? (2) What are the best predictors of AGB in phylogenetic diversity and phylogenetic structure as vegetation regenerates? (3) How does vegetation regeneration drive phylogenetic diversity and phylogenetic structure to influence AGB changes? We hypothesized that in the temperate zone, a higher AGB is caused by greater evolutionary diversity. However, this is dependent on the vegetation regeneration process. This study contributes to our current understanding of the crucial developmental pathways associated with BEF and imparts valuable insights for plantation forest construction.

## 2 Methods

### 2.1 Research Sites and Approach

**FIGURE 1:**
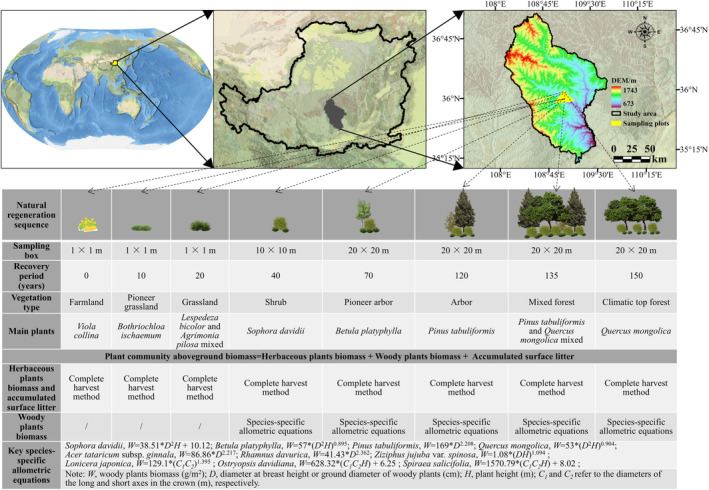
Ziwuling research sites in the Chinese Loess Plateau.

The Ziwuling Nature Reserve was selected as the research site for this study, which is located within the geographical coordinates of 34°50′–36°50′ N and 107°30′–109°40′ E in the Loess Plateau of north‐Central China (Figure 1). The area experienced an extensive war between 1842 and 1866, which resulted in decreased in human activity and farmland abandonment after harvest. Subsequently, natural vegetation recovery commenced in the area and persisted for over 150 years, resulting in the establishment of a dense forest landscape characterized by a natural secondary forest (Tian, Zhang, Xu, et al. 2022). In this study, we employed a “space‐for‐time” approach from 2020 to 2021 to establish standardized sample plots representing eight distinct recovery periods for typical vegetation types. A total of 48 sites were included, with six sites allocated for each recovery period: 0 year (Farmland, 1 × 1 m sampling box); 10 year (Pioneer grassland, 1 × 1 m sampling box); 20 year (Grassland, 1 × 1 m sampling box); 40 year (Shrub, 10 × 10 m sampling box); 70 year (Pioneer arbor, 20 × 20 m sampling box is); 120 year (Arbor, 20 × 20 m sampling box); 135 year (Mixed forest, 20 × 20 m sampling box); and 150 year (Climatic top forest, 20 × 20 m sampling box; Figure 1).

Since 1954, numerous studies on the natural vegetation regeneration in the Ziwuling have found that the local vegetation adheres to the general pattern of vegetation succession in temperate deciduous broad‐leaved forest areas (Chen 1958; Zhao et al. 2015; Cui et al. 2020). Additionally, Lv (2009) and Lu et al. (2016) reported the establishment of a *Bothriochloa ischaemum* community ~10 years after agricultural land abandonment. Deng et al. (2013) observed the evolution of the vegetation into a grassland community with shrubs. Concurrently, Shangguan and Wang (2008) found that a *Sophora davidii* shrub community appeared after the grassland stage, and Liang et al. (2010) found that a Pioneer arbor (*Betula platyphylla* forest) appeared around the 70th year. Research conducted by Zou, Liu, and Wang (2002), Fan, Wang, and Guo (2006), and Tian et al. (2006) demonstrated that the transition to the arbor stage and climatic top forest in the region requires > 120–150 years. In conclusion, recovery timing was determined by synthesizing these reports with similar land‐use histories and findings from relevant forestry departments. Sites had similar stand conditions and soil texture (elevation variation < 200 m; Tian, Zhang, Xu, et al. 2022). At each sample site, we documented species occurrence using name and number. Species names underwent meticulous verification using The Plant List (http://www.theplantlist.org) and Plants of the World Online (http://powo.science.kew.org). Life form and ecological type were ascertained based on the Flora of China (Li 2007), including consideration of plant habitat conditions.

### 2.2 Phylogenetic Reconstruction

**FIGURE 2:**
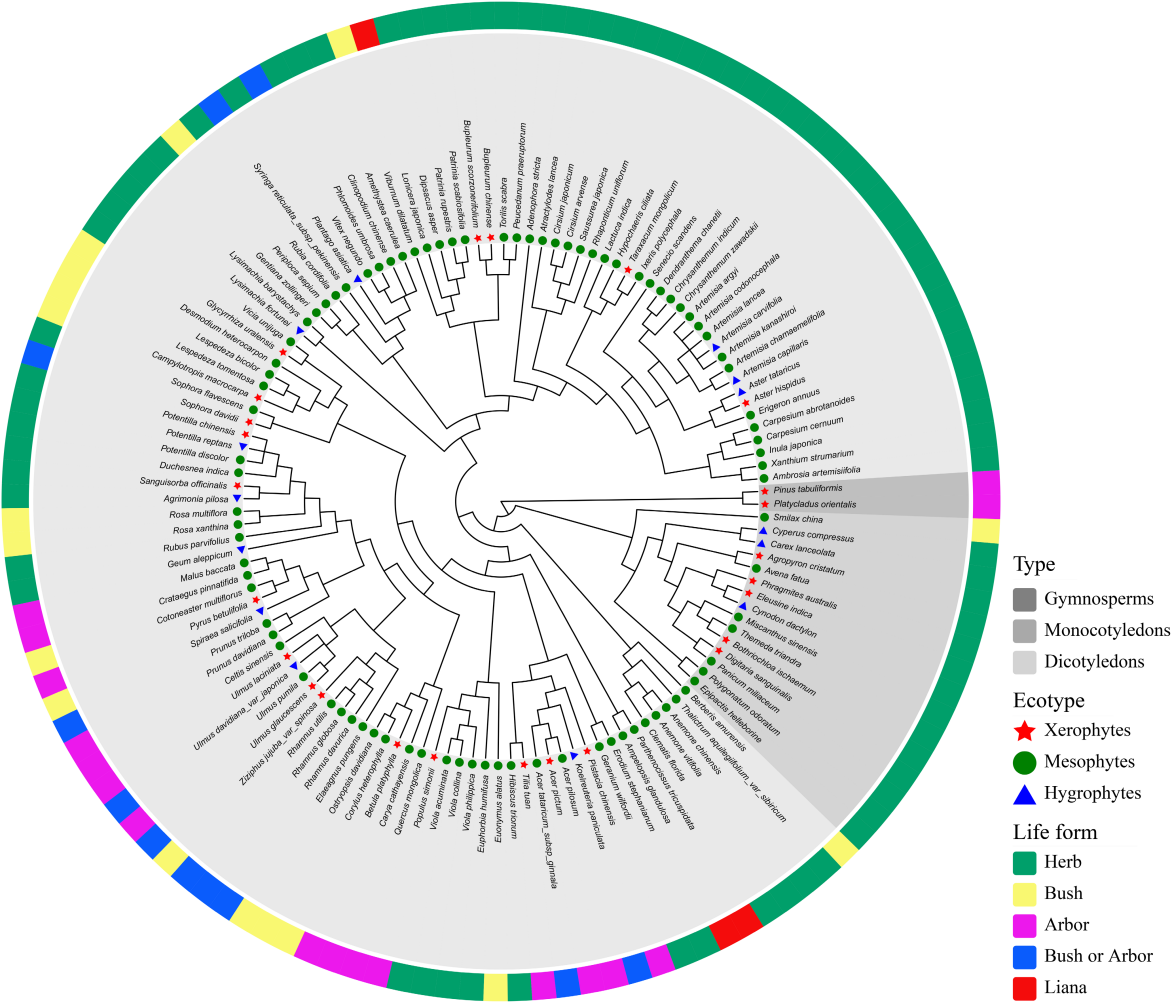
Phylogenetic relationship of plants sampled from plots representing 150 years of natural vegetation regeneration progress.

We used a recently published species‐level phylogenetic tree generated by Jin and Qian (2022) as a basis for generating a phylogeny for the different recovery stages of seed plant species. Our phylogenetic tree represents an enhanced and extended iteration of the megaphylogeny GBOTB (Smith and Brown 2018), which uses a dataset of 79,881 taxa from GenBank and backbone derived from the Open Tree of Life (Version 9.1). Consequently, the tree provides the most comprehensive and current time‐calibrated megaphylogeny available for spermatophytes at the species level. The phylogenetic relationships of all species in our study could be resolved by the TPL nomenclature standardization system megatree (GBOTB.extended.TPL.tre). We utilized the V. PhyloMaker2 software (build. nodes.1 and scenario 3) to prune the phylogenetic tree, retaining only species for which we possessed corresponding data (Figure 2).

Phylogenetic diversity: Faith's phylogenetic diversity (PD; Faith 1992) is a widely employed metric for quantifying the phylogenetic diversity of assemblages. This method measures the cumulative length of all phylogenetic branches connecting the species within an assemblage and captures the range of phylogenetic characteristics. Phylogenetic species richness (PSR) is a phylogenetic indicator derived by multiplying the number of species by the phylogenetic species variability in the studied community. Phylogenetic species evenness (PSE) is a phylogenetic indicator that considers the relative abundances of species, ranging from 1 (i.e., equal abundances of distantly related species) to 0 (i.e., unequal abundances of closely related species; Ali and Yan 2018). These three indicators provide a complete and easily understood phylogenetic measure elucidating community evolutionary diversity (Helmus et al. 2007).

Phylogenetic structure: The net relatedness (NRI) and net nearest taxon (NTI) indices are often employed to characterize the ecological processes of the community assembly and used in combination with phylogenetic diversity (Webb, Ackerly, and Kembel 2008; Li et al. 2022). The NRI describes the genealogical structure of species development in a community as a whole, whereas the NTI defines the genealogical patterns of communities composed of closely related species (Swenson et al. 2007).

An NRI or NTI > 0 indicates that a community contains an aggregated phylogenetic structure of species and a tendency for closely related species to co‐occur in the same community, which highlights the dominant role of environmental filtering; if NRI or NTI = 0, the phylogenetic structure is stochastic and fits the neutral theory; an NRI or NTI < 0 indicates a divergent phylogenetic structure, that a community contains more distantly related species, and that competitive exclusion is the dominant force shaping community structure (Webb et al. 2002).
(1)

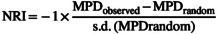



(2)

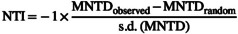




In Equations (1) and (2), mean phylogenetic distance (MPD) in a community represents the average phylogenetic distance among all species, while mean nearest taxon distance (MNTD) represents the average distance to the nearest phylogenetic taxon among all species. MPDob and MNTDob are the observation values of MPD and MNTD, respectively; MPDrandom and MNTDrandom are random zero‐model (*n* = 999) means, and s.d. represents the standard deviation of the distribution obtained from the zero‐model. The zero model was created by randomly selecting a group of species that were seen 999 times in each sample and using the whole phylogeny as the sampling pool.

### 2.3 Plant Community Aboveground Biomass

AGB is the sum of herbaceous and woody plant biomass, along with accumulated surface litter. Three sampling boxes were randomly selected at the sample site to collect herbaceous plants biomass and accumulated surface litter (1 × 1 m and 31.7 × 31.7 cm sampling boxes, respectively) using the complete harvest method (Ravindranath and Ostwald 2007; Tian et al. 2023). Woody plants biomass was calculated as the sum of individual tree biomass in the plot using species‐specific allometric equations (Figure 1). In forest ecosystems, this method is widely used to quantify biomass based on relative growth relationships through allometric equations (Vorster et al. 2020). The allometric equations used in this study were obtained from Gang, Xiang, and Birong (2014), Li (2010), Liu (2021), Luo, Fang, and Hu (2017), and Tian, Li, et al. (2021).

### 2.4 Statistical Analysis

Polynomial fitting was employed to linearly fit the trend of phylogenetic diversity over recovery time. The impact of vegetation recovery on the phylogenetic structure and aboveground biomass of plant communities across various periods was examined using one‐way ANOVA, followed by post hoc testing (Duncan's test, *p* < 0.05). Data organization and analysis were performed using Excel 2019 (Microsoft Corp, Redmond, WA, USA) and SPSS (version 21.0; IBM Corp, Armonk, NY, USA). Subsequently, the data were visualized using Origin 2021b (OriginLab, Northampton, MA, USA). Data normality was assessed using the Shapiro–Wilk (S–W) test, while homogeneity of variances was examined using Levene's test. In cases where the data deviated from a normal distribution, a log‐ or power‐function transformation was applied to ensure homogeneity of variances. Furthermore, generalized linear mixed models (GLMMs) were computed at different levels, with “recovery stage” included as a random factor to account for historical, climate, or stochastic factors associated with various recovery periods (Bates et al. 2015). The model performance of the GLMMs was evaluated based on the Akaike Information Criterion (AIC) in addition to the *R* ^*2*^ *m* and *R* ^*2*^ *c* statistics (Barton 2020), providing marginal and conditional estimates of model fit with and without the random factor, respectively. Finally, the relationship between plant phylogeny and AGB was investigated using variance partitioning analysis (VPA) and partial least squares path modeling (PLS‐PM; Sanchez 2013; Tian et al. 2019). GLMM, VPA, and PLS‐PM analyses were conducted using R 4.2.1 (R Foundation for Statistical Computing, Vienna, Austria).

## 3 Results

### 3.1 Vegetation Characteristics

Overall, 128 spermatophyte species were found belonging to 99 genera in 39 families (Figure 2). This comprised 2 gymnosperm species (2 genera, 2 families); 126 species of angiosperms, among which 111 were dicotyledon species (82 genera, 32 families); 15 monocotyledons (15 genera, 5 families), indicating the domination of dicotyledons. In general, plants can be categorized into three ecological types based on their tolerance to water conditions: xerophytes, mesophytes, and hygrophytes. Among the species recorded in this study, 89 (69.53%) were mesophytes, 25 (19.53%) were xerophytes, and 14 (10.94%) were hygrophytes.

Life form is the morphological manifestation of plant adaptability to the external environment. This study identified five major life forms: herbs, bushes, arbors, lianas, and bushes or arbors. Herb species dominated the community with 79 (61.72%), followed by 18 bush (14.06%), 17 arbor (13.28%), 11 bush or arbor (8.60%), and only 3 liana (2.34%) species. To understand the evolutionary characteristics of the investigated community during different phases of succession, plant life forms were further classified into herbaceous and woody plants (79 and 49 species, respectively) for analysis, according to a synopsis of the Chinese flora (Li 2007) and actual survey.

### 3.2 Phylogenetic Diversity

**FIGURE 3:**
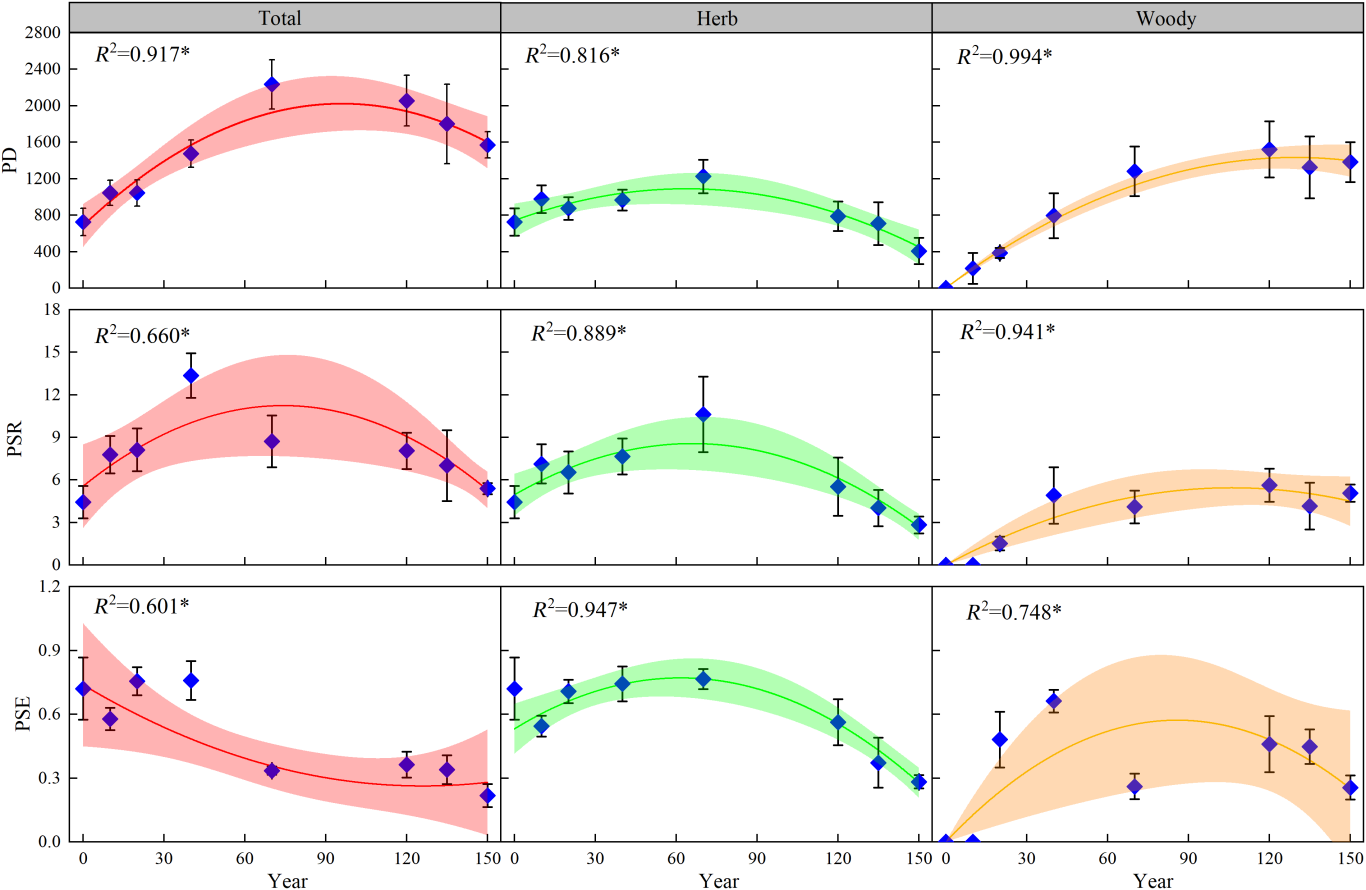
Linear regression was used to analyze the relationships between phylogenetic diversity and the periods of natural vegetation regeneration (*p < 0.05). Dark‐colored lines represent the fitted linear model for phylogenetic diversity and recovery stage, and the whiskers represent the 95% confidence intervals of these linear models. “Total,” “Herb,” and “Woody” represent the total, herbaceous, and woody species of the community, respectively. PD, phylogenetic diversity; PSE, phylogenetic species evenness ; PSR, phylogenetic species richness.

We examined alterations in phylogenetic diversity from three species perspectives: total community, herb, and woody. The correlation between the recovery stages and three phylogenetic diversity indices of the plant community exhibited a singular peak throughout the 150‐year period (excluding total community PSE; Figure 3). Herbaceous plant diversity peaked at ~60 years after the initiation of vegetation recovery (TVR; in years). Total community diversity and woody plants peaked at ~90 and 120 TVR, respectively. However, total community PSE values diminished with vegetation recovery and gradually stabilized after 90 TVR.

### 3.3 Phylogenetic Structure

**FIGURE 4:**
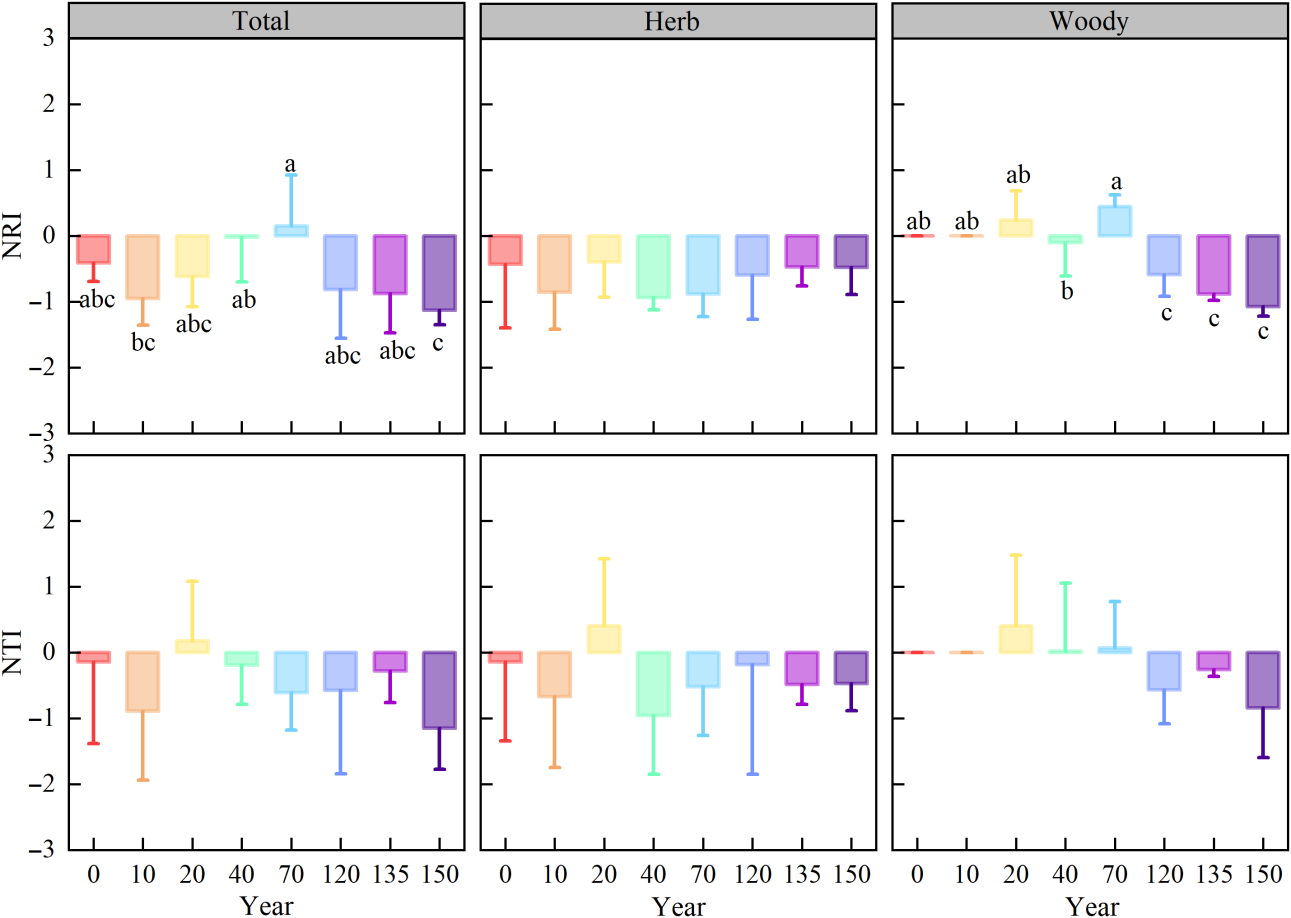
Phylogenetic structure patterns of the net relatedness index (NRI) and nearest taxon index (NTI). Lowercase letters (a–c) denote significant differences (*p* < 0.05) within each variable among the distinct recovery periods. “Total,” “Herb,” and “Woody” represent the total, herbaceous, and woody species of the community, respectively.

Statistically significant alterations were detected in the NRI (*p* < 0.05) at both the total community and woody plant levels throughout the course of vegetation recovery (Figure 4). Specifically, an initial increase followed by a subsequent decreasing trend was observed. Thus, the total community exhibited a transition from phylogenetic dispersion to aggregation, and a return to dispersion. In contrast, the woody plants showed a pattern of phylogenetic randomness transitioning to aggregation, and a returning to dispersion. Furthermore, the NRI index of herbaceous plants and NTIs for total, herbaceous, and woody plants showed no significant changes across various stages of vegetation recovery.

### 3.4 Plant Community Aboveground Biomass

**FIGURE 5:**
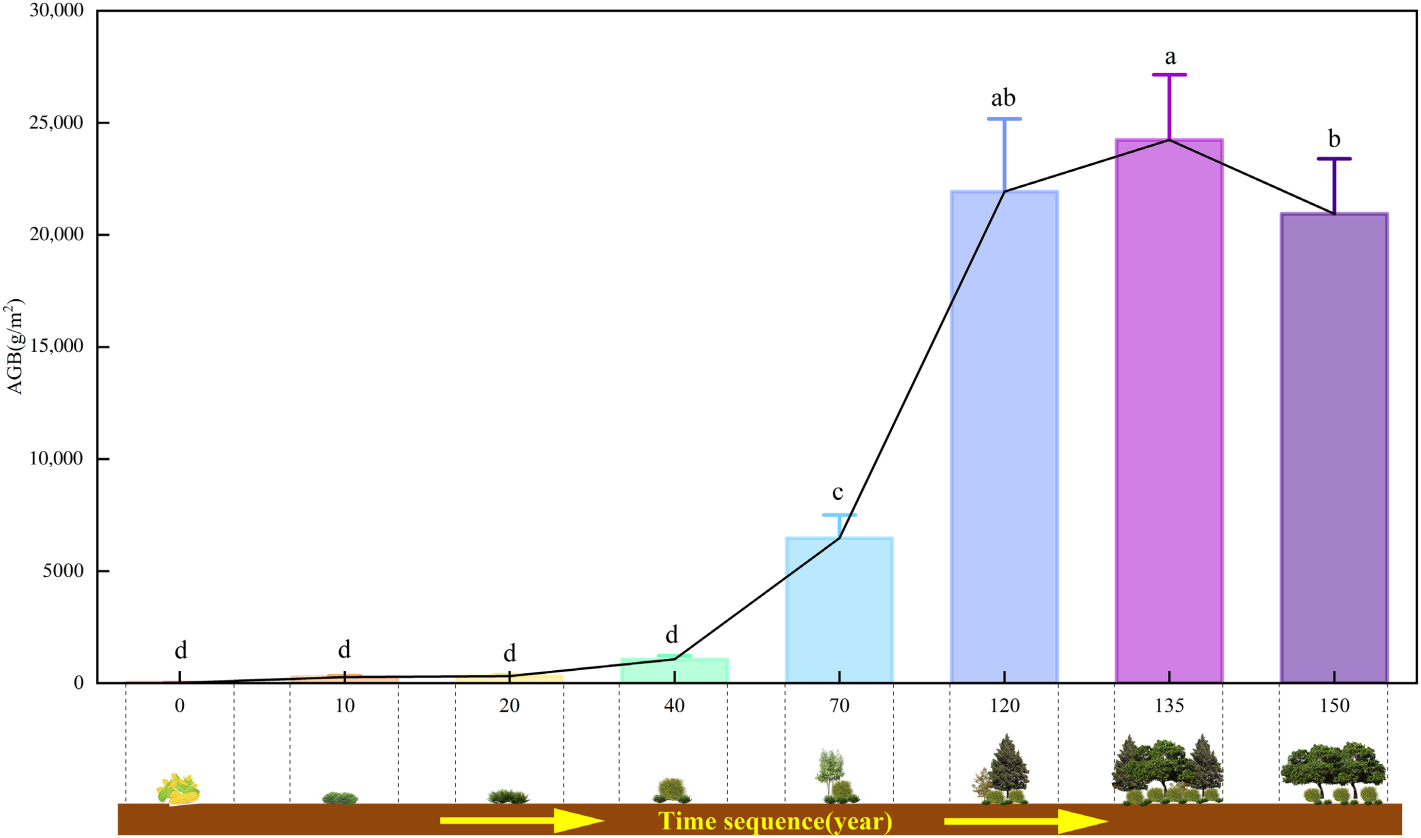
Total aboveground biomass of the investigated plant community during 150 years of natural vegetation regeneration. Lowercase letters (a–d) represent significant (*p* < 0.05) differences within each variable among the different recovery periods.

AGB increased drastically with the progression of vegetation recovery (Figure 5). A peak was observed at ~135 TVR (24233.88 ± 2914.85 g/m^2^), with the greatest increase observed from 70 to 120 TVR, and second greatest increase from 40 to 70 TVR. When the plant community reached the top stage (150 TVR), AGB decreased significantly.

### 3.5 Relationship Between Phylogenetic Diversity and Total Aboveground Biomass

Distinct outcomes were observed for the three levels within the GLMMs (Table 1). The explained variation (*R* ^2^m) was the highest in the total community and woody levels, followed by the herbaceous level. Vegetation recovery was important in all cases (*R* ^2^c > *R* ^2^m), particularly for herbs. Evolutionary diversity features significantly affecting AGB at the total community level were PD and PSR. The herbaceous level was mainly correlated with PD. The woody level was mainly correlated with PD, PSE, and NRI. PD was therefore the main and most consistent predictor of AGB.

**TABLE 1:** Results of the GLMMs of aboveground biomass and five predictors of evolutionary diversity, using recovery stage as the random factor, as well as model parameters for the three levels.

	Total	Herb	Woody
AIC	346.363	376.792	355.850
*R* ^2^m	0.640	0.033	0.577
*R* ^2^c	0.999	0.987	0.996
PD	11.949*	2.658*	2.27*
PSR	−7.296*	−1.526	0.441
PSE	−0.059	−1.165	3.524*
NRI	1.61	−0.88	−2.266*
NTI	0.439	1.148	0.174

*Note:* AIC is the value of the Akaike Information Criterion. *R* ^2^m and *R* ^2^c show the marginal/conditional estimates of model fits (without/with the random factor) as estimated. The numbers in the main part of the table are *t* values of the coefficients with significance levels (*p < 0.05) for each explanatory variable. “Total,” “Herb,” and “Woody” represent the total, herbaceous, and woody species of the community, respectively.

Abbreviations: NRI, net relatedness index; NTI, nearest taxon index; PD, phylogenetic diversity; PSE, phylogenetic species evenness; PSR, phylogenetic species richness.

**FIGURE 6:**
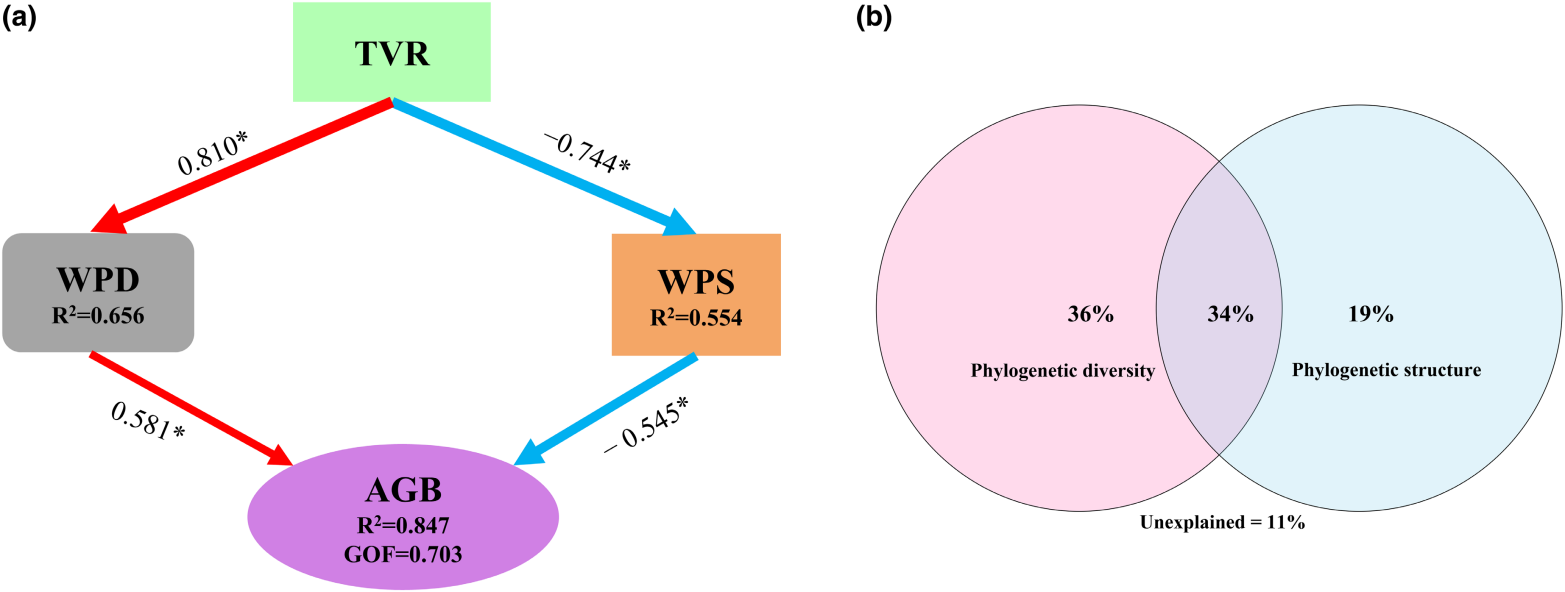
Partial least squares path analysis (PLS‐PM) and variance partitioning analysis (VPA) for the aboveground biomass (AGB) of the plant community, showing the relationships between evolutionary diversity and AGB. (a) PLS‐PM analysis of woody levels. (b) VPA of woody levels. The path coefficients on the arrows indicate the magnitudes and directions of the effects, with positive and negative values indicating positive and negative effects represented by red and blue lines, respectively. (*p < 0.05). TVR, time since vegetation recovery (in years); WPD includes phylogenetic diversity and phylogenetic species evenness; WPS is the net relatedness index.

Additionally, we conducted a PLS‐PM and VPA at the woody level. The PLS‐PM application demonstrated a significant relationship between the evolutionary diversity of the plant community and pathways related to AGB, which involved PD and phylogenetic structure influenced by vegetation recovery (Figure 6). The results showed that vegetation recovery had a positive impact on AGB through increased PD, and phylogenetic structure was a negative determinant. Woody level explains 84.7% of the AGB variance (GOF = 0.703; Figure 6a). Furthermore, the VPA showed that the overall explanatory power of woody plant phylogeny for AGB changes (after long‐term forest recovery) was similar to the PLS‐PM results. The combined effects of PD and phylogenetic structure exhibited the greatest explanatory power for AGB (34.0%), with PD and phylogenetic structure independently explaining 36% and 19%, respectively.

## 4 Discussion

### 4.1 Phylogenetic Diversity, Structure, and Aboveground Biomass in Different Recovery Periods

The predominance of mesophytic plants and coexistence of multiple life forms observed in this study was consistent with the pattern of secondary forest succession, suggesting that plants have a strong adaptive capacity to handle environmental changes and improve resource utilization through spatial differentiation of ecological niches within a plant community (Finegan 1984; Donato, Campbell, and Franklin 2012). The PD and PSR values observed during vegetation recovery are consistent with the single‐peak model reported by Chai et al. (2016). The different times at which herbaceous and woody plants reached peak species richness are related to the deterministic process of the ecological niche. In this process, plants in a community help different species to coexist by exhibiting different spatiotemporal use patterns (Tian, Zhang, Xu, et al. 2022). The PSE gradually decreases in the late successional stage (after 40 TVR). It is possible that in highly productive forests, weak competitors will be outcompeted by competitors with high resource utilization due to evolutionary relatedness, as predicted by the competitive exclusion effect (Kunwar et al. 2021).

Our phylogenetic structure analysis revealed that the phylogenetic structure shifted from dispersion (or randomness) to aggregation and reverted to dispersion during the 150‐year vegetation recovery process. This phenomenon was markedly visible in woody plant scenarios, which are influenced by the transformation from abiotic to biotic community drivers (Chun and Lee 2019). Environmental filtering is responsible for phylogenetic aggregation, whereas competition results in excessive phylogenetic dispersion (Swenson et al. 2012). These results align with those of previous studies conducted by Cardinale et al. (2007) and Reich et al. (2012), which demonstrated an increase in complementary effects between species over time and an accompanying decrease in selection effects. During the revegetation period, herbaceous plants mostly exhibited dispersed and stochastic neutral processes. We speculate that good microhabitats remained in place, which weakened environmental filtering. Further, the area had a large number of species and a variety of dispersal patterns that allowed herbaceous plants to regularly colonize new patches (Tian, Xu, et al. 2021).

Our findings illustrated the tremendous potential of vegetation recovery to increase the carbon sink capacity (Feng et al. 2013; Chen et al. 2021) and highlight the common interests of biodiversity conservation and carbon sequestration (Rahman et al. 2021). Additionally, we discovered that the AGB of mixed forests comprising evergreen conifers (*Pinus tabuliformis*) and deciduous broadleaves (*Quercus mongolica*) was higher than that of pure forests (*Quercus mongolica*) when vegetation recovery reached the top stage. This result is consistent with the findings of Lu et al. (2016), providing further theoretical evidence that mixed planting is advantageous for enhancing the carbon sink function of plants and mitigating climate change (Huang et al. 2018; Tang et al. 2022).

### 4.2 Relationships Among Phylogenetic Diversity, Phylogenetic Structure, and Aboveground Biomass

The diversity–biomass link depends heavily on environmental factors, in addition to the net influence of species composition on biomass (Ammer 2019; Cai, Li, and Jin 2020; Wen et al. 2022). PD values were found to be the most reliable predictors of AGB at all three levels as per GLMM analysis. The results of this analysis highlight the value of PD values for gauging how ecological processes may affect future outcomes (Flynn et al. 2011; Mourya, Bargali, and Bargali 2019). By comparing *R* ^2^m values, we discovered that woody plants had the greatest influence on vegetation AGB, and PSE was highly significant. This shows that metrics related to species evenness are important factors in regulating AGB (Chun, Ali, and Lee 2020). Meanwhile, *R* ^2^c indicated that the explanatory power of the stochastic factor for AGB during long‐term vegetation recovery was ~40% at the total community and woody levels. This highlights the tendency of certain highly abundant species to provide most of the AGB, which provides evidence of environmental filtering of phylogenetically conserved traits (Lemanski, Williams, and Winfree 2022).

In addition, we considered the pathways of environmental variation driving phylogenetic diversity and phylogenetic structure on AGB (as represented by vegetation recovery processes). The PLS‐PM results showed that 84.7% of the variance in AGB could be explained by the evolutionary variety of woody plants. This is related to the selection effect (Grime 1998), particularly for species with strong ecological functions (Satdichanh et al. 2019). The significant positive correlation between woody plant PD and AGB, and the considerable negative correlation between the phylogenetic structure index and AGB provide strong support for the findings of Cadotte, Cardinale, and Oakley (2008) and Genung, Schweitzer, and Bailey (2014), among others. Notably, the neutral process of herbaceous plants may influence the phylogenetic structure at the total community level, which subsequently results in a nonsignificant impact of the phylogenetic structure at the total community level on AGB.

In conclusion, our findings suggest that the ecological niche complementation process and the selection effect do not contradict each other over time. That is, rich PD facilitates the sampling effect and causes the community AGB to increase under the environmental filter. As ecological succession progresses to later stages, competitive exclusion can fragment community phylogenetic structure and potentially facilitate the mechanism of niche complementation and a subsequent AGB increase. This is consistent with the evolutionary viewpoint (Molina‐Venegas et al. 2021), in addition to classic community dynamics that assumes abiotic processes predominate early in succession and biotic processes gain prominence in later stages (Muscarella et al. 2016). This aids in the comprehension of the characteristics and mechanisms of spatiotemporal changes in biodiversity and ecosystem function and is necessary for the correct prediction of future climate change‐related impacts.

This study presents several key findings: (1) Changes in evolutionary diversity driven by natural vegetation regeneration can significantly affect plant community AGB; (2) PD and phylogenetic structure contribute differently to AGB; (3) phylogenetically close species can drive AGB changes in different periods; and (4) phylogenetically distant species can raise the upper limit of AGB in the same period, because they exhibit different overlapping ecological niches and functional diversity under selection pressure as a result of evolutionary divergence (Cadotte 2013). Notably, our study did not incorporate disturbance history and other random factors, as the excluded variables might be contingent on the specific stage of vegetation recovery (Chu et al. 2019). Therefore, a more reliable depiction of AGB changes during long‐term forest recovery in temperate zones is grounded in the evolutionary diversity of woody plants. Additionally, we propose a replanting protocol that suggests using combinations of distantly related species and various life types to optimize the ecological functions of planted forests (Di Sacco et al. 2021).

## 5 Conclusions

Our findings imply that biodiversity conservation and carbon sequestration may be mutually beneficial. A few highly abundant species tend to provide most of the AGB during revegetation, with PD being the main predictor for AGB. The evolutionary diversity of woody plants is more accurate in explaining AGB changes during long‐term vegetation restoration in temperate zones. Under environmental filtering, phylogenetically close species can promote AGB changes, while phylogenetically distant species can increase the upper limit of AGB during the same period. Therefore, combinations of distantly related species and different life form types are favorable for improving the ecological function of plantation forests. As the relationship between plant diversity and AGB is influenced by many factors and processes, future studies should include environmental factors (i.e., climate and soil) and a broader range of biodiversity (i.e., fauna and microorganisms) in their analyses.

## Author Contributions

**Qilong Tian:** conceptualization (lead), data curation (equal), investigation (equal), writing – original draft (lead), writing – review and editing (equal). **Xiaoping Zhang:** conceptualization (equal), data curation (equal), funding acquisition (equal), investigation (equal), project administration (equal), resources (equal), writing – review and editing (equal). **Miaoqian Wang:** formal analysis (equal), investigation (equal). **Jie He:** formal analysis (equal), investigation (equal). **Xiaoming Xu:** formal analysis (equal), investigation (equal). **Liang He:** investigation (equal). **Haijie Yi:** investigation (equal). **Haojia Wang:** investigation (equal).

## Conflicts of Interest

The authors declare no conflicts of interest.

## Data Availability

The original contributions presented in this study are included in the article/supporting information. Further inquiries can be directed to the corresponding author.
